# Targeting myocyte-specific enhancer factor 2D contributes to the suppression of cardiac hypertrophic growth by miR-92b-3p in mice

**DOI:** 10.18632/oncotarget.20759

**Published:** 2017-09-08

**Authors:** Zhi-Qin Hu, Jian-Fang Luo, Xue-Ju Yu, Jie-Ning Zhu, Lei Huang, Jing Yang, Yong-Heng Fu, Tao Li, Yu-Mei Xue, Ying-Qing Feng, Zhi-Xin Shan

**Affiliations:** ^1^ Guangdong Cardiovascular Institute, Guangdong Provincial Key Laboratory of Clinical Pharmacology, Guangzhou, China; ^2^ Guangdong General Hospital, Guangdong Academy of Medical Sciences, Guangzhou, China; ^3^ Department of Forensic Pathology, Zhongshan School of Medicine, Sun Yat-Sen University, Guangzhou, China; ^4^ School of Medicine, South China University of Technology, Guangzhou, China

**Keywords:** microRNA-92b-3p, cardiac hypertrophy, cardiomyocyte, MEF2D

## Abstract

The role of microRNA-92b-3p (miR-92b-3p) in cardiac hypertrophy was not well illustrated. The present study aimed to investigate the expression and potential target of miR-92b-3p in angiotensin II (Ang-II)-induced mouse cardiac hypertrophy. MiR-92b-3p was markedly decreased in the myocardium of Ang-II-infused mice and of patients with cardiac hypertrophy. However, miR-92b-3p expression was revealed increased in Ang-II-induced neonatal mouse cardiomyocytes. Cardiac hypertrophy was shown attenuated in Ang-II-infused mice received tail vein injection of miR-92b-3p mimic. Moreover, miR-92b-3p inhibited the expression of atrial natriuretic peptide (ANP), skeletal muscle α-actin (ACTA1) and β-myosin heavy chain (MHC) in Ang-II-induced mouse cardiomyocytes *in vitro*. Myocyte-specific enhancer factor 2D (MEF2D), which was increased in Ang-II-induced mouse hypertrophic myocardium and cardiomyocytes, was identified as a target gene of miR-92b-3p. Functionally, miR-92b-3p mimic, consistent with MEF2D siRNA, inhibited cell size increase and protein expression of ANP, ACTA1 and β-MHC in Ang-II-treated mouse cardiomyocytes. Taken together, we demonstrated that MEF2D is a novel target of miR-92b-3p, and attenuation of miR-92b-3p expression may contribute to the increase of MEF2D in cardiac hypertrophy.

## INTRODUCTION

Hypertension, genetic polymorphisms, loss of cardiomyocytes following ischaemic damage and altered cardiac metabolism are known as the main causes of pathological cardiac hypertrophy [1, 2]. At the early stage of pathological hypertrophy, the increased size of cardiomyocytes is initially a compensatory mechanism, however, the sustained hypertrophy may eventually lead to dilated cardiomyopathy, arrhythmia, heart failure and even sudden death [3–5]. Currently, no efficient therapeutic approaches are available for the treatment of cardiac hypertrophy. Understanding the molecular mechanisms associated with cardiac hypertrophy and the transition to heart failure is crucial to identify new therapeutic targets to prevent or reverse cardiac hypertrophy and heart failure.

MicroRNAs (miRNAs), endogenous 20–23-nucleotide noncoding RNAs, play important roles in the process of cardiac hypertrophy [6]. For example, microRNA-1, -16 and microRNA-181b were down-regulated in cardiac hypertrophy, and *in vitro* over-expression of them resulted in the reduced size of cardiomyocytes [7–9]. By contrast, microRNA-208a and microRNA-195 were up-regulated in cardiac hypertrophy, which were sufficient to drive pathological cardiac growth when over-expressed in transgenic mice, respectively [10, 11].

MicroRNA-92b-3p (miR-92b-3p) was shown elevated in peripheral blood of patients with chronic systolic heart failure [12]. MiR-92b-3p regulates Mef2 levels through a negative-feedback circuit during Drosophila muscle development [13], but the role of miR-92b-3p in cardiac hypertrophy has not been well understood.

Transcription factors (TFs) are nuclear proteins that bind to the promoter regulatory elements to activate/repress the downstream gene expression. Some hypertrophic TFs, such as myocyte enhancer factor 2 (MEF2), NK family of transcription factor 2.5 (Nkx2.5), GATA4, nuclear factor of activated T-cells (NFAT), and Ca^2+^/cAMP response element-binding protein (CREB) [14–16], have been known implicated as signal-responsive mediators of the cardiac transcriptional program in cardiac hypertrophy.

MEF2-binding A/T-rich DNA sequences have been identified within the promoter regions of cardiac genes, including α-MHC, myosin light chain (MLC)2v, skeletal α-actin, cardiactroponin T, -C, and -I [17, 18]. MEF2D, a key regulatory protein for cardiac development, is a primary MEF2 factor expressed in the adult heart. MEF2D mediates pressure overload and beta-chronic adrenergic stimulation-induced cardiac remodeling [19, 20]. Previous studies reported that MEF2D was as a target of miR-122, -218 in cardiac myxoma cells [21, 22].

In this study, we observed a significant decrease of miR-92b-3p in mouse and human hypertrophic myocardium. Enforced enhancement of miR-92b-3p ameliorated angiotensin II (Ang-II) infusion-induced cardiac hypertrophy in mice. Our results demonstrated that miR-92b-3p negatively regulated MEF2D expression by directly targeting the 3′untranslated region (UTR) of MEF2D mRNA. Either miR-92b-3p mimic or MEF2D siRNA could efficiently inhibit Ang-II-induced hypertrophy in neonatal mouse ventricular cardiomyocytes (NMVCs). Our data suggest that MEF2D is a novel target of miR-92b-3p in myocardial hypertrophy, and enhancement of miR-92b-3p expression may be protective against myocardial hypertrophy.

## RESULTS

### Decreased expression of miR-92b-3p in the hypertrophic myocardium

**Figure 1:**
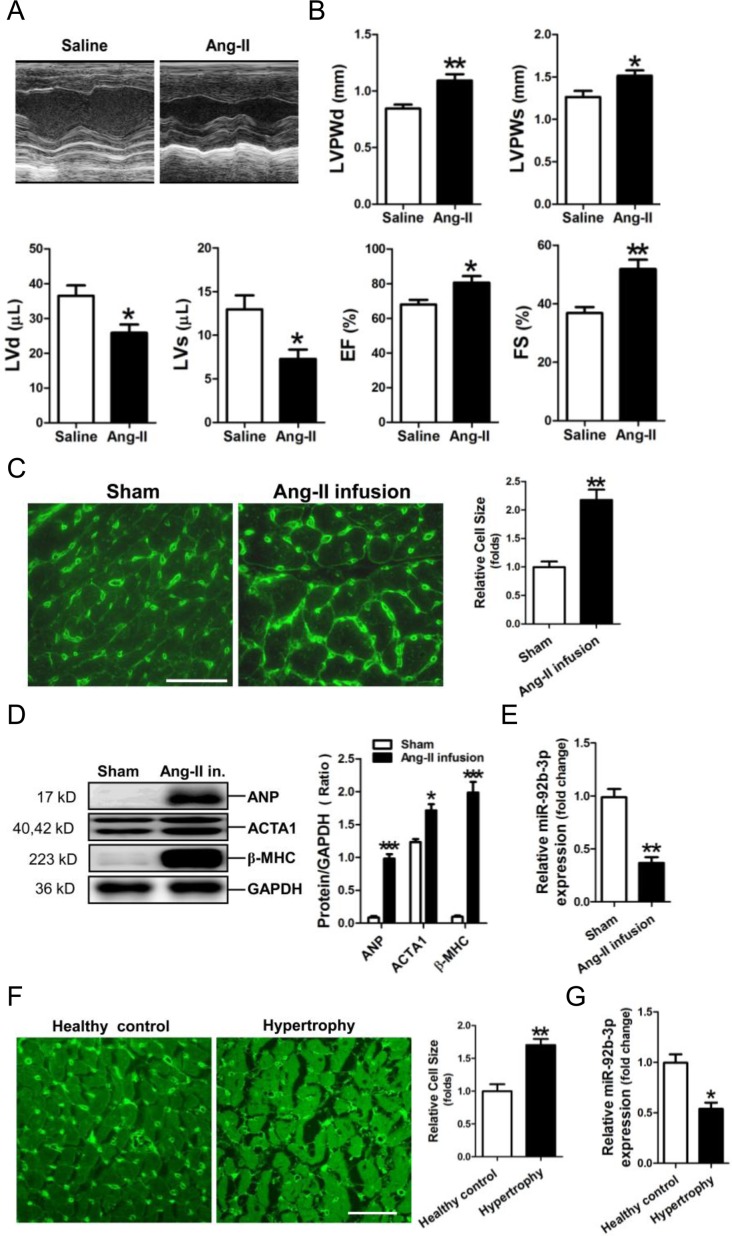
Down-regulation of microRNA-92b-3p (miR-92b-3p) in the hypertrophic myocardium **(A)** Representative echocardiographs of mouse hearts. **(B)** The representative variables of echocardiograph assay in mice, including LVPWd, LVPWs, LVd, LVs, EF and FS. Data are shown as mean ± sem, ^*^p < 0.05, ^**^p < 0.01, ^***^p < 0.001 *vs* saline group. N = 6–8. **(C)** WGA staining assay of the hypertrophic myocardium of a mouse model of Ang-II-infusion-induced hypertrophy. The scale bar is 50 μm. **(D)** Expressions of ANP, ACTA1 and β-MHC in mouse myocardium by Western blot assay. **(E)** Expression of miR-92b-3p in mouse myocardium by RT-qPCR assay. Data are shown as mean ± sem, ^*^p < 0.05, ^**^p < 0.01, ^***^p < 0.001 *vs* sham group. N = 5–8. **(F)** WGA staining assay of the hypertrophic myocardium of patients with cardiac hypertrophy. The scale bar is 50 μm. **(G)** Expression of miR-92b-3p in human myocardium by RT-qPCR assay. Data are shown as mean ± sem, ^*^p < 0.05, ^**^p < 0.01 *vs* healthy control. N = 8.

An animal model of hypertrophy was established in mice with Ang-II infusion. Echocardiography was performed to reveal cardiac structure and function changes in Ang-II-infused mice (Figure 1A). The thickened LV walls (LVPWd, LVPWs) and decrease in the LV volume (LVd, LVs) were observed in the hypertrophic mouse hearts. In addition, the compensatory increases of ejection fraction (EF) and fractional shortening (FS) were shown markedly increased in Ang-II-infused mice (Figure 1B).

WGA staining showed that the cell size of cardiomyocytes was significantly increased in the myocardium of the Ang-II-infused mice (Figure 1C). Results of Western-blotting showed that the hypertrophy-associated genes, including ANP, ACTA1 and β-MHC, were significantly increased in mouse myocardium subjected to Ang-II treatment (Figure 1D). RT-qPCR assay revealed that miR-92b-3p was significantly decreased in the myocardium of mice received Ang-II infusion (Figure 1E).

Moreover, the size of cardiomyocytes was markedly increased in the myocardium of patients with cardiac hypertrophy (Figure 1F). Consistently, RT-qPCR results showed that miR-92b-3p was also decreased in human hypertrophic myocardium (Figure 1G).

### MiR-92b-3p attenuates Ang-II-induced cardiac hypertrophy *in vivo*

To further demonstrate the potential role of miR-92b-3p in Ang-II-induced cardiac hypertrophy, we determined whether restoring miR-92b-3p expression via tail vein injection of miR-92b-3p agomir could exert protective effect on the cardiac hypertrophy.

**Figure 2:**
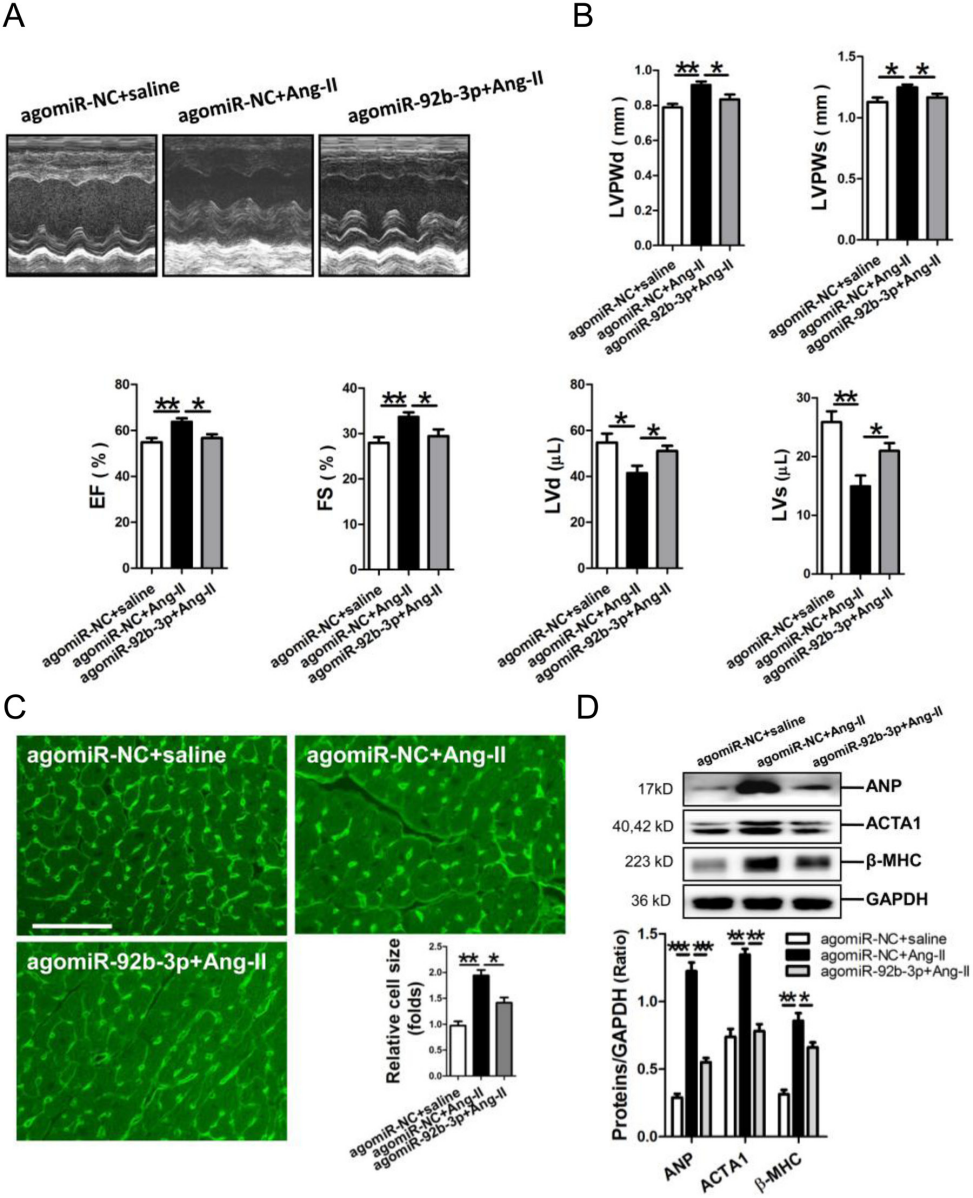
Phenotype of cardiac hypertrophy of Ang-II-infused mice with enforced expression of miR-92b-3p **(A)** Representative echocardiographs of mouse hearts. **(B)** The representative variables of echocardiograph assay in mice, including LVPWd, LVPWs, LVd, LVs, EF and FS. **(C)** WGA staining assay of the myocardium of Ang-II-infused mice with enforced expression of miR-92b-3p. The scale bar was 50 μm. **(D)** Expressions of ANP, ACTA1 and β-MHC in mouse myocardium by Western blot assay. Data are shown as mean ± sem, ^*^p < 0.05, ^**^p < 0.01, ^***^p < 0.001. N = 6–8.

Results of echocardiography showed that the thickened LV walls (LVPWd, LVPWs) and decrease in the LV volume (LVd, LVs) were reversed in Ang-II-infused mice received miR-92b-3p agomir injection. Additionally, miR-92b-3p agomir could significantly attenuate the compensatory increases of EF and FS in Ang-II-infused mice (Figure 2A, 2B). The size of cardiomyocytes was markedly increased in the myocardium of Ang-II-infused mice, but which could be reversed by the enforced expression of miR-92b-3p (*p*<0.05, *p*<0.01, respectively) (Figure 2C). Meanwhile, Western blot results demonstrated that expression of ANP, ACTA1 and β-MHC in mouse myocardium in response to Ang-II infusion was also suppressed by miR-92b-3p delivery (*p*<0.05, *p*<0.01, *p*<0.001, respectively) (Figure 2D).

### MiR-92b-3p attenuates the hypertrophic growth in cardiomyocytes

**Figure 3:**
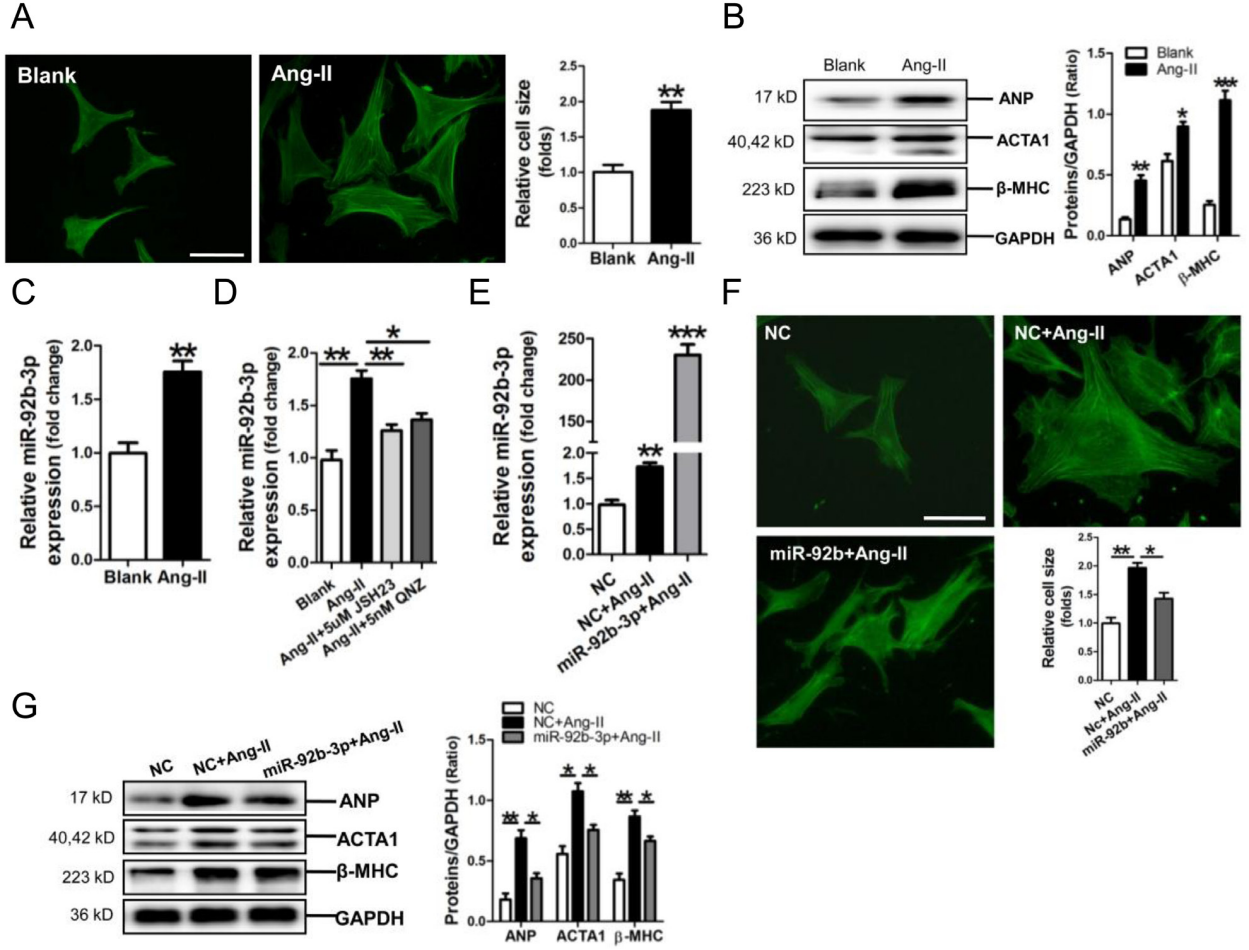
Up-regulation of microRNA-92b-3p (miR-92b-3p) and the suppressive effect of miR-92b-3p on expressions of ANP, ACTA1 and β-MHC in Ang-II-induced mouse cardiomyocytes **(A)** FITC-phalloidin staining of Ang-II-induced hypertrophic NMVCs. **(B)** Expressions of ANP, ACTA1 and β-MHC in Ang-II-treated NMVCs by Western blot assay. **(C)** Expression of miR-92b-3p in NMVCs by RT-qPCR assay. Data are shown as mean ± sem, ^*^p < 0.05, ^**^p < 0.01, ^***^p < 0.001 *vs* blank control. N = 3. **(D)** MiR-92b-3p expression in Ang-II-induced NMVCs with pre-treatment with the NF-κB inhibitor JSH23 or QNZ, respectively. Data are shown as mean ± sem, ^*^p < 0.05, ^**^p < 0.01. N = 3. **(E)** Determination of miR-92b-3p level in NMVCs. Data are shown as mean ± sem, ^**^p < 0.01, ^***^p < 0.001 *vs* NC group. N = 3. **(F)** FITC-phalloidin staining of Ang-II-induced NMVCs with overexpression of miR-92b-3p. The scale bar is 50 μm. **(G)** Expressions of ANP, ACTA1 and β-MHC in Ang-II-induced NMVCs with overexpression of miR-92b-3p by Western blot assay. Data are shown as mean ± sem, ^*^p < 0.05, ^**^p < 0.01. N = 3.

We established a cell model of Ang-II-induced mouse cardiomyocyte hypertrophy, resulting in significant increase of cell size and ANP, ACTA1 and β-MHC protein expression (*p*<0.05, *p*<0.01, *p*<0.001, respectively) (Figure 3A, 3B). Meanwhile, miR-92b-3p was observed upregulated in Ang-II-treated NMVCs (*p*<0.01) (Figure 3C). We previously demonstrated that NF-κB signaling was activated in cardiomyocytes exposed to Ang-II [23]. In the present study, RT-qPCR results revealed that NF-κB P65 inhibitor JSH23 or QNZ could efficiently abolish the upregulation of miR-92b-3p by Ang-II in NMVCs (*p*<0.05, *p*<0.01, respectively) (Figure 3D).

Results of RT-qPCR assay confirmed that miR-92b-3p was efficiently transfected into NMVCs (Figure 3E), and FITC-phalloidin staining demonstrated that the increase of cell size of Ang-II-treated NMVCs was markedly reversed by miR-92b-3p mimic (Figure 3F). Consistently, the up-regulations of ANP, ACTA1 and β-MHC protein in Ang-II-induced NMVCs were significantly suppressed by over-expression of miR-92b-3p (Figure 3G).

### Verification of MEF2D as a target gene of miR-92b-3p

**Figure 4:**
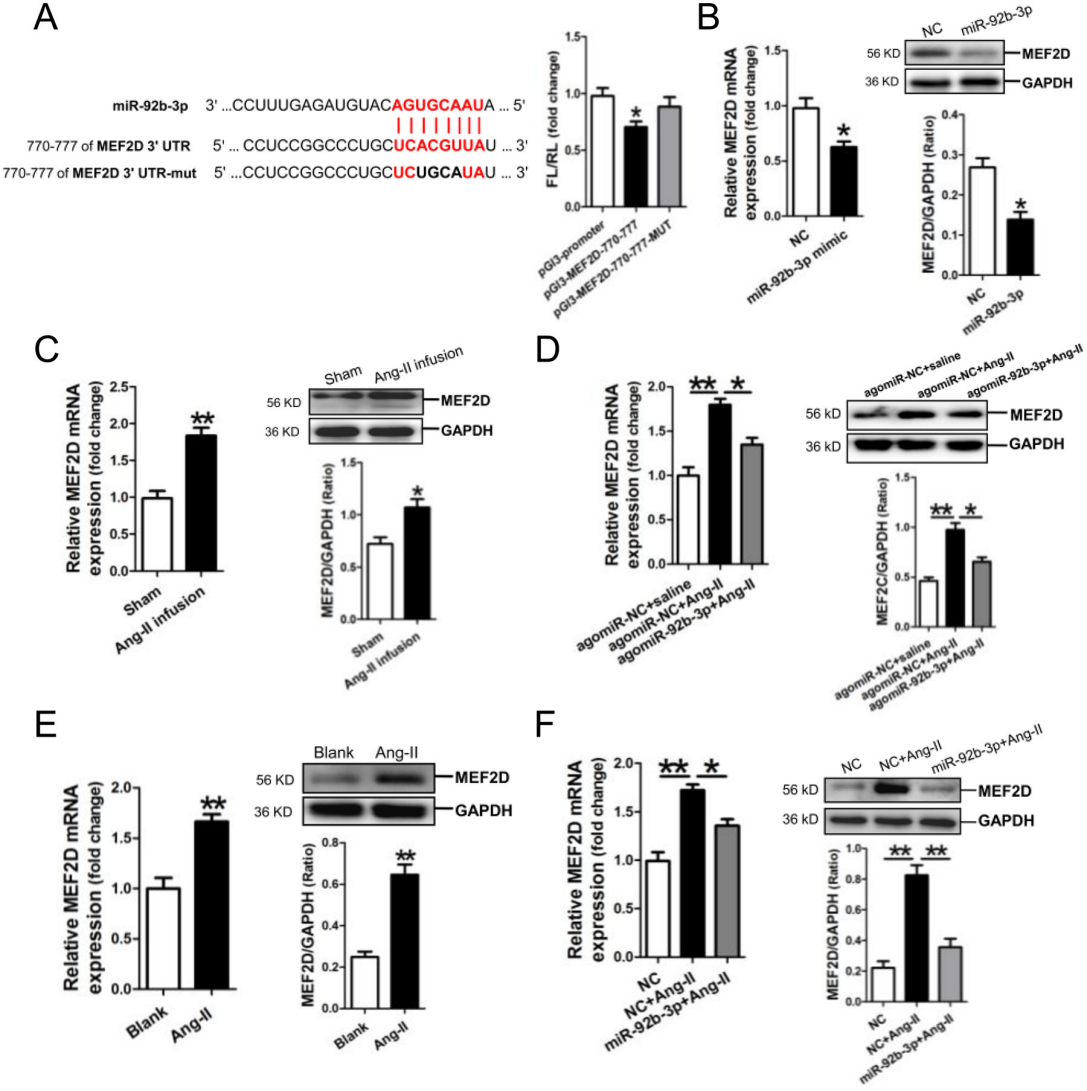
MicroRNA-92b-3p (miR-92b-3p) negatively modulates MEF2D expression **(A)** Verification of MEF2D as a target gene of miR-92b-3p by the dual luciferase reporter system. Predicted miR-92b-3p seed matches to the sequence in the 3′UTR of MEF2D mRNA. The seed sequence of miR-92b-3p is UAACGUGA, and the complementary nucleotide sequences are shown in red words. Data are shown as mean ± sem, ^*^p <0.05 *vs* pGl3-promoter vector control, N=3. **(B)** MRNA and protein expressions of MEF2D in miR-92b-3p-modified NMVCs by RT-qPCR and Western blot assay, respectively. MRNA and protein expressions of MEF2D in the myocardium of mice received Ang-II infusion only **(C)** and in the myocardium of mice received Ang-II combined with agomiR-92b-3p delivery **(D)**. Data are shown as mean ± sem, ^*^p <0.05, ^**^p <0.01. N=6-8. MRNA and protein expressions of MEF2D in Ang-II-treated NMVCs **(E)** and in Ang-II-induced NMVCs with overexpression of miR-92b-3p **(F)**. Data are shown as mean ± sem, ^*^p <0.05, ^**^p <0.01. N=3.

Analysis of the databases Mirdb (www.mirdb.org) and TargetScan-Vert (www.targetscan.org) showed that MEF2D was a potential target gene of miR-92b-3p. The matching positions for miR-92b-3p within 3′-UTR of the targeted mRNA is shown in Figure 4A. The dual luciferase assay demonstrated that miR-92b-3p significantly reduced the luciferase activity through binding the site of 770–777 of MEF2D 3′-UTR (*p*<0.05) (Figure 4A).

Then, we examined the expression of MEF2D in NMVCs after transfection with miR-92b-3p mimic. Compared with the negative scramble control, mRNA and protein expression of MEF2D were significantly decreased in miR-92b-3p-modified NMVCs (*p*<0.05, respectively) (Figure 4B).

Next, we detected the expression of MEF2D in Ang-II-induced cardiac hypertrophy *in vivo* and *in vitro*. MRNA and protein expression of MEF2D were markedly increased in the myocardium of Ang-II-infused mice (Figure 4C), but was reversed in the myocardium of mice received Ang-II combined with agomiR-92b-3p treatment (Figure 4D). Moreover, mRNA and protein expression of MEF2D were also shown increased in Ang-II-treated NMVCs (Figure 4E), but was reversed in Ang-II-induced NMVCs with enforced expression of miR-92b-3p (Figure 4F).

### MiR-92b-3p and MEF2D siRNA attenuate the hypertrophic phenotype in cardiomyocytes

**Figure 5:**
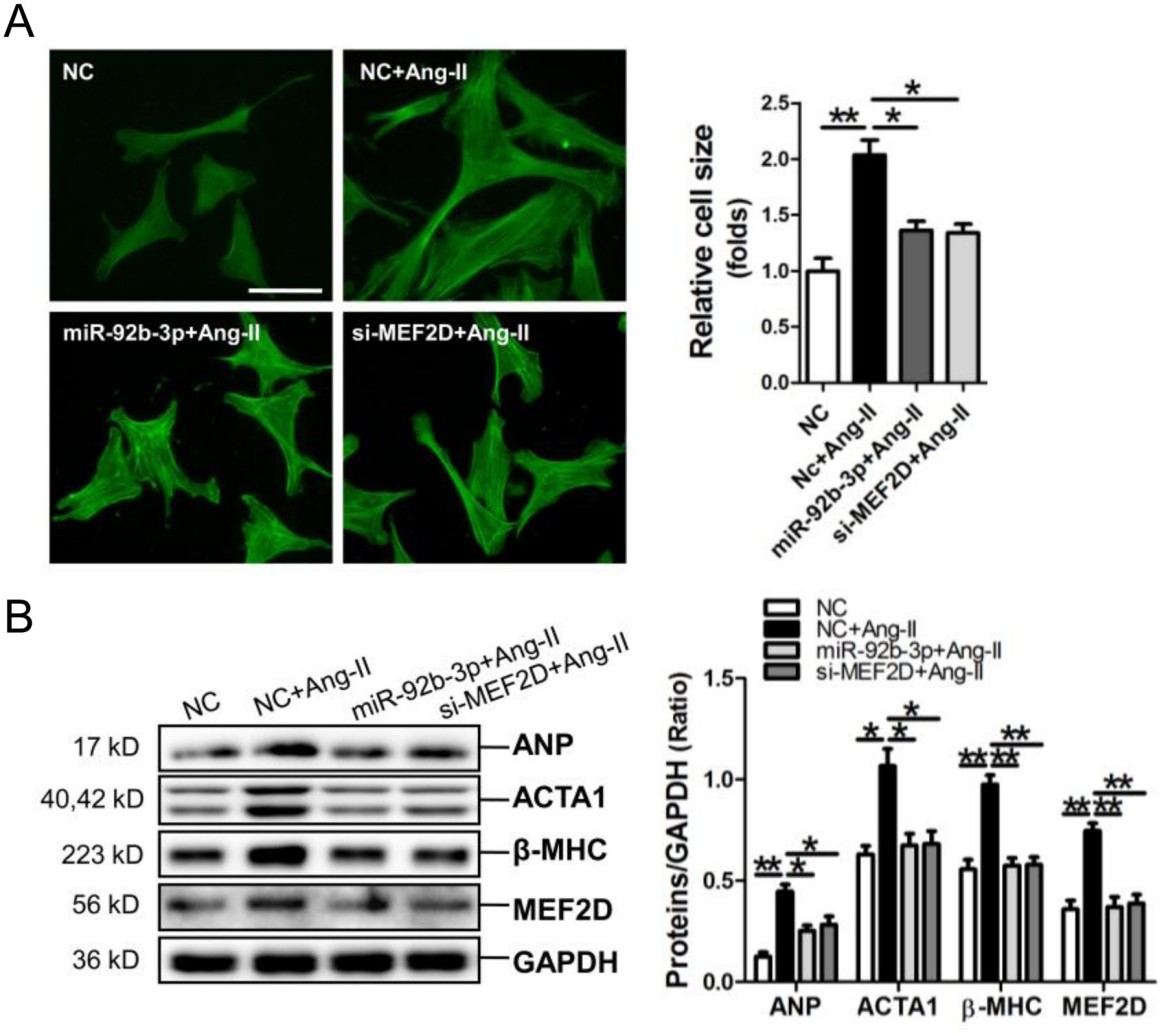
MicroRNA-92b-3p (miR-92b-3p) suppresses the hypertrophic phenotype of NMVCs *in vitro* **(A)** Morphologies of Ang-II-treated NMVCs as revealed by FITC-phalloidin staining. The scale bar is 50 μm. **(B)** ANP, ACTA1, β-MHC and MEF2D protein expression in NMVCs was detected by Western blot assay. Data are shown as mean ± sem, ^*^p<0.05, ^**^p<0.01. N=3.

MiR-92b-3p mimic and MEF2D siRNA were transfected into NMVCs, followed by FITC-phalloidin staining assay and determining the expressions of hypertrophy-related genes. Results of FITC-phalloidin staining showed that miR-92b-3p mimic and MEF2D siRNA could efficiently suppress the increase of cell size of Ang-II-induced NMVCs (Figure 5A). Consistently, the Western blot results showed that the up-regulations of ANP, ACTA1, β-MHC and MEF2D by Ang-II in NMVCs could be reversed by miR-92b-3p mimic and MEF2D siRNA, respectively (Figure 5B).

## DISCUSSION

In the present study, miR-92b-3p was observed significantly decreased in Ang-II infusion mouse model of cardiac hypertrophy. Additionally, decrease of miR-92b-3p was also observed in the myocardium of patients with hypertrophy. Contrast to the previous report that miR-92b-3p was elevated in peripheral blood of patients with chronic heart failure [12], our present data indicated that the increased circulating miR-92b-3p may not derived from the myocardium of patients with cardiac hypertrophy.

We verified that miR-92b-3p was upregulated in Ang-II-induced hypertrophic mouse cardiomyocytes in this study. Previous reports suggested the important role for the NF-κB signaling in cardiac hypertrophy [24–26]. Similarly, we previously demonstrated that NF-κB signaling was activated in mouse cardiomyocytes exposed to Ang-II treatment [23]. In the present study, we confirmed that NF-κB P65 inhibitor JSH23 or QNZ could efficiently abolish the upregulation of miR-92b-3p in Ang-II-induced NMVCs. These data revealed that NF-κB signaling mediates the upregulation of miR-92b-3p in Ang-II-induced mouse cardiomyocytes. Nevertheless, the mechanism underlying the down-regulation of miR-92b-3p in human and mouse hypertrophic myocardium warrants further investigation.

Since our data showed that miR-92b-3p was markedly down-regulated in the myocardium of Ang-II-infused mice. Thereafter, we delivered miR-92b-3p agomir via tail vein to increase miR-92b-3p level in mouse myocardium to further investigate the role of miR-92b-3p in Ang-II-induced cardiac hypertrophy. As expected, the compensatory increase of heart function was restored and the altered cardiac structures were also reversed by miR-92b-3p in Ang-II-infused mice. Consistently, miR-92b-3p also significantly attenuated the upregulations of ANP, ACTA1 and β-MHC by Ang-II *in vivo* and *in vitro*. Therefore, our data demonstrated a protective role of miR-92b-3p in cardiac hypertrophy.

Activations of the MEF2 family members (MEF2A, 2B, 2C and 2D) of transcription factors play important roles in the regulation of cardiac hypertrophy and remodeling [27–29]. Transcriptional factor MEF2 integrates Ca^++^/calmodulin-dependent signaling pathway and is a common downstream target of various hypertrophic stimuli in heart in the context of cardiac hypertrophy [30]. The transcriptional activity of MEF2 can also be enhanced by interaction with other transcription factors such as GATA4 and NFAT [31].

MEF2D was verified to participate in pressure overload and beta-chronic adrenergic stimulation-induced cardiac remodeling [19, 20]. Consistently, the present study showed that MEF2D was significantly increased in the hypertrophic myocardium of Ang-II-infused mice and in Ang-II-treated mouse cardiomyocytes. Moreover, knockdown of MEF2D by miR-92b-3p or siRNA targeting MEF2D inhibited Ang-II-induced cardiomyocyte hypertrophy *in vivo* and *in vitro*, respectively.

Our current study has provided several lines of evidence to support the notion that miR-92b-3p inhibits cardiac hypertrophy through targeting MEF2D. First, the *in silico* prediction indicated that MEF2D was a potential target of miR-92b-3p, and the dual luciferase assay revealed that miR-92b-3p specifically bound to the 770-777 site in the 3′-UTR of MEF2D. Additionally, miR-92b-3p mimic inhibited MEF2D expression at both mRNA and protein levels in mouse cardiomyocytes. Importantly, the upregulations of MEF2D mRNA and protein by Ang-II in cardiomyocytes could be reversed by miR-92b-3p *in vivo* and *in vitro*. Moreover, in parallel with the findings with MEF2D siRNA, over-expression of miR-92b-3p decreased the cell size of cardiomyocytes and inhibited the expressions of ANP, ACTA1 and β-MHC in Ang-II-induced mouse cardiomyocytes.

Taken together, our data have demonstrated that miR-92b-3p is down-regulated in cardiac hypertrophy, and miR-92b-3p ameliorates cardiac hypertrophic responses *in vivo* and *in vitro*. Our data has also revealed that miR-92b-3p inhibits hypertrophic phenotype in cardiomyocytes through down-regulation of transcription factor MEF2D expression. Therefore, the present study suggests that miR-92b-3p might be a potential target for prevention and treatment of cardiac hypertrophy.

## MATERIALS AND METHODS

### Ethics statement

Male C57BL/6 mice weighing 20±3 g and 1- to 3-d old newborn C57BL/6 mice (License number SCXK (YUE) 2004–0011, Department of Experimental Animal Research Center, Sun Yat-sen University, Guangzhou, China) were used in the current studies. The adult mice were housed under a 12-h light/dark cycle under pathogen-free conditions and with free access to standard mouse chow and tap water. This study conformed to the Guide for the Care and Use of Laboratory Animals published by the US National Institutes of Health (8th Edition, National Research Council, 2011). The present program was also approved by the research ethics committee of Guangdong General Hospital (the approval number: No. GDREC2010093A).

The human ventricular samples were stored and donated by the tissue bank of the Department of Forensic at Sun Yat-sen University in Guangzhou, China.

### Animal studies

According to previously described methods, we established a mouse Ang-II (1.46 mg/kg/d, 14 d) infusion model of cardiac hypertrophy [32]. Mice were anesthetized through the intraperitoneal application of sodium pentobarbital (50 mg/kg), followed by implantation of the Ang-II mini-osmotic pump (alzet model 2002, Cupertino, CA, USA). The adequacy of anesthesia was confirmed by the absence of reflex response to foot squeeze. Body temperature was maintained at 37±0.5°C during surgery. At the end of the experiments, mice were killed with the intraperitoneal injection of an overdose of sodium pentobarbital (200 mg/kg).

To investigate the effect of miR-92b-3p on Ang-II-induced hypertrophy *in vivo*, 24 C57BL/6 mice were randomized into 3 groups: 1) agomiR-negative control (NC)+saline, 2) agomiR-NC+Ang-II (NC agomir with Ang-II infusion), and 3) agomiR-92b-3p+Ang-II (miR-214-3p agomir with Ang-II infusion). All agomirs were purchased from (Guangzhou RiboBio, Guangzhou, China). The amount of 20 nmol NC agomir or 20 nmol miR-92b-3p agomir was delivered into each mouse via tail vein injection at 4 interval time points within 14 d.

### Echocardiography

Left ventricular (LV) function variables were assessed by transthoracic echocardiography 2 weeks after the mini-osmotic pump implant surgery. After the induction of light general anesthesia, the mice received transthoracic two-dimensional (2D) guided M-mode echocardiography (Technos, MP, 8.5-MHz transducer). From the cardiac short axis (papillary level), the end-systolic and end-diastolic LV posterior wall diameters (LVPWs and LVPWd), the end-systolic and end-diastolic LV volume (LVs and LVd), ejection fraction (EF), and fractional shortening (FS) were measured. Echocardiographic measurements were averaged from at least three separate cardiac cycles.

### Wheat germ agglutinin (WGA) staining

Mice were sacrificed with an overdose of sodium pentobarbital (200 mg/kg, ip) at the end of experiments. The mouse heart was excised, and the LV myocardium was fixed overnight in 10% formalin. Samples were embedded in paraffin and cut into 4 μm thick sections. Tissue sections were mounted on the regular glass slides and stained with 1.0 mg/mL Alexa Fluor^®^ 488 conjugate of WGA solution (MolecularProbes, Eugene, Oregon, USA) to demonstrate the size of cardiomyocytes in mouse or human ventricular myocardium.

### Primary culture of neonatal mouse ventricular cardiomyocytes and treatments

Neonatal mouse ventricular cells (NMVCs) were isolated from the hearts of 1–3-d-old newborn C57BL6 mice as described previously [33]. The newborn mice were killed by cervical dislocation. Isolated NMVCs were plated onto 12-well plates and maintained for 48 h in DMEM/F-12 supplemented with 10% FBS (Gibco, New York, NY). NMVCs were incubated with 10^−8^ M Ang-II for 48 h to induce the hypertrophic phenotype. NMVCs were treated with NF-κB inhibitor JSH23 (5 μM) and QNZ (5 nM), respectively. NMVCs were also transfected with 50 nM scramble or miR-92b-3p mimic, or 50 nM siRNA targeting MEF2D (Ribobio, Guangzhou, China) by oligofectamine reagent (Invitrogen, Carlsbad, CA), respectively.

### FITC-phalloidin staining

Cultured NMVCs were fixed in 3.7% formaldehyde and permeabilized in 0.1% Triton X-100 for 10 min, respectively, followed by incubation with blocking solution for 40 min and subsequently with FITC-phalloidin (10 μg/ml, Sigma-Aldrich) at 37°C. Confocal micrographs were obtained using a Leica SP5 confocal microscope (Leica, Mannheim, Germany). Cell size (total area) was quantified using MiVnt imaging software (Weiyu, Zhuhai, China). The average cell size of about 50 NMVCs from 3 different views was determined. Three independent repeats of FITC-phalloidin staining were performed in this study.

### Quantitative miRNA and mRNA measurements

Quantitative reverse-transcription PCR (qRT-PCR) for miR-92b-3p was performed on cDNA generated from 0.5 μg total RNA according to the manufacturer's protocol (Ribobio, China). For the detection of mRNA expression of coding genes, the first-strand cDNAs were generated from 1.5 μg total RNA using a mixture of oligo (dT)_15_ and random primers with superscriptreverse transcriptase (Invitrogen, Carlsbad, CA). To normalize RNA content, U6 was used for miR-92b-3p template normalization and GAPDH was used for coding genes template normalization. PCR was performed with the ViiA7 Quantitative PCR System (Applied Biosystems, Carlsbad, CA). The 2^−ΔΔCt^ method was used to calculate relative expression levels of the concerned miRNAs and coding genes. PCR primers for miRNAs, U6 and coding genes are shown in Supplementary Table 1.

### Western blot analysis

The amount of 40-50 μg protein prepared from mouse myocardium or NMVCs was used in a standard western blot analysis. The polyvinylidene fluoride (PVDF) membrane binding sample protein was incubated with a high affinity anti-ANP antibody (1:500 dilution), anti-ACTA1 antibody (1:500 dilution), anti-β-MHC antibody (1:1000), anti-MEF2D antibody (1:1000)(Abcam, Cambridge, MA). An anti-GAPDH antibody (1:2000) (Santa CruzBiotechnology, USA) was used to detect the level of GAPDH as an internal control. Protein was visualized using the ECL Plus detection system (GE Healthcare, Waukesha, WI).

### Dual luciferase assay for MEF2D target identification

According to our previous report [34], the recombinant luciferase reporter plasmid containing the potential miR-92b-3p binding site sequences of MEF2D gene was constructed. Using a site-directed mutagenesis kit (TransGen, Beijing, China), the miR-92b-3p binding site sequence GTGCAAT was replaced with GTCGTTT to construct the corresponding recombinant luciferase reporter plasmid containing the mutant potential miR-92b-3P binding sequences. Human embryonic kidney (HEK) 293 cells (3×10^5^ cells per well in the 12-well plate) were cotransfected with 200 ng of recombinant luciferase reporter plasmid, 50 nM miR-92b-3p mimic, and 20 ng of pRL-TK plasmid as an internal control (Promega, Madison, WI). Activities of firefly luciferase (FL) and Renilla luciferase (RL) were measured 24 h after transfection. The relative ratio of the FL/RL was used to indicate the suppression of MEF2D by miR-92b-3p.

### Statistical analysis

The data are presented as the means±s.e.m. In each experiment, all determinations were performed at least in triplicate. Statistical significance between two measurements was determined by the two tailed unpaired Student's *t* test, and among groups, it was determined by one-way ANOVA. A value of *p*< 0.05 was considered to be significant.

## SUPPLEMENTARY MATERIALS FIGURES AND TABLES


