# Supplementary material for: Targeting myocyte-specific enhancer factor 2D contributes to the suppression of cardiac hypertrophic growth by miR-92b-3p in mice
Source: Oncotarget. 2017 Sep 8;8(54):92079–89. doi: 10.18632/oncotarget.20759 (PMC5696165; doi:10.18632/oncotarget.20759)
Supplement: Supplementary file 1 [file oncotarget-08-92079-s001.pdf]

Targeting myocyte-specific enhancer factor 2D contributes to the suppression of cardiac hypertrophic growth by miR-92b-3p in mice

SUPPLEMENTARY MATERIALS

Supplementary Table 1: Primers used in qRT-PCR assay

Gene	Sequence (5′- 3′)	Product size (bp)
MEF2D	F, CCTCCTTACCAGCCTTCAGT<br>R, CAGGGATGAGGTTGCTGAGA	198
GAPDH	F, CAAGAAGGTGGTGAAGCAGG<br>R, CCACCCTGTTGCTGTAGCC	200
miR-92b-3p	RT, GTCGTATCCAGTGCGTGTCGTGGAGT<br>CGGCAATTGCACTGGATACGACGGAGGCCG<br>F, GTCCGCTATTGCACTCGTCCCGGCCTCC<br>R, GTGCGTGTCGTGGAGTC	66
U6	RT, GTCGTATCCAGTGCGTGTCGTGGAGT<br>CGGCAATTGCACTGGATACGAC<br>F, GTCCGCGTGCTCGCTTCGGCAGC<br>R, GTGCGTGTCGTGGAGTC	160
